# Constancy bias: When we “fill in the blanks” of unattended or forgotten stimuli

**DOI:** 10.3758/s13414-019-01838-w

**Published:** 2019-08-19

**Authors:** Dock Duncan, Stefan Van der Stigchel

**Affiliations:** Department of Experimental Psychology, Helmholtz Institute, Utrecht University, Utrecht, the Netherlands

**Keywords:** Change detection, Inattentional blindness, Working memory, Attention

## Abstract

Our ability to form predictions about the behavior of objects outside our focus of attention and to recognize when those expectations have been violated is critical to our survival. One principle that greatly influences our beliefs about unattended stimuli is that of constancy, or the tendency to assume objects outside our attention have remained constant, and the next time we attend to them they will be unchanged. Although this phenomenon is familiar from research on inattentional blindness, it is currently unclear when constancy is assumed and what conditions are adequate to convince us that unattended stimuli have likely undergone a change while outside of our attentional spotlight. Using a simple change-detection task, we sought to show that unattended stimuli are strongly predisposed to be perceived as unchanging when presented on constant, unchanging backgrounds; however, when stimuli were presented with significant incidental visual activity, participants were no longer biased towards change rejection. We found that participants were far more likely to report that a change had occurred if target presentation was accompanied by salient, incidental visual activity. We take these results to indicate that when an object is not represented in working memory, we use environmental conditions to judge whether or not these items are likely to have undergone a change or remained constant.

## Introduction

Most of what we see we ignore. When we gaze upon a scene, our brain immediately goes about arranging the flood of visual information into coherent objects in space (Treisman & Gelade, 1980). The process of moving from unrefined feature information to complete perceptual objects is the central question of Feature Integration Theory, and, as remarkable a feat as this quick and automatic process is, perhaps equally impressive is our rapid identification of what information is important for our immediate goals, and the subsequent direction of attention towards a subset of this visual information while relegating the remaining majority of our visual environment to a fallow inattentional state (Treisman, 2006).

Although at any moment we are ignoring a large portion of our visual environment, attention is not a prerequisite for perception (Koch & Tsuchiya, 2007), and the information we ignore does not disappear from our vision. Instead, this unattended visual information must still be processed and arranged into coherent objects in a systematic and rule-based way (Braun & Sagi, 1990; Rosenholtz et al., 2012; Treisman & Gelade, 1980). Some characteristics of this inattentional system of perception are known, such as our implicit ability to learn statistical regularities of scenes without attention (Chun & Jiang, 1999; Treisman, 2006; Turk-Brown et al., 2005) and our remarkable ability to quickly extract gist information from rapidly presented stimuli (Chen & Treisman, 2009; Evans & Treisman, 2005; Greene & Oliva, 2009; Li et al., 2002; Rensink, 2004). There remains much to be learned about how we perceive in the absence of attention; and given the limited scope of attention, understanding inattention will lead to a better understanding of how the majority of our sensory information is processed in the brain. Despite this, researchers have paid relatively less attention to inattentional visual processes than to the principles governing objects within our focus (Duncan, 1984; Posner et al., 1980; Treisman, 1998, 2006), making inattentional visual principles an area ripe for further investigation.

One known principle of inattentional vision is that of constancy (i.e., the tendency to perceive objects in a stable environment as constant and unchanging over time). Constancy is most familiar from the striking results of inattentional blindness studies, wherein changes to objects outside of our attention are extremely difficult to detect (Irwin, 1996; Rensink et al., 1997; for a review see Simons & Ambinder, 2005). The results of these studies have led to the assertion that attention is critical for change detection (Rensink et al., 1997). Taken a step further, because unattended stimuli are still represented in our perceptual experience and because attention is a necessary prerequisite for change detection, it follows that objects outside of our immediate attention must necessarily be perceived as constant and unchanging. Constancy is therefore an important principle for unattended stimuli: if you are not paying attention to something, assume it has not changed.

Several questions remain open in regard to constancy, such as: What happens when objects seem likely to have undergone a change while outside of our attention? Or: are we biased to assume that unmemorized items remain constant in the same way that we assume unattended items do? One research paradigm that has approached this second question comes in the form of the flicker paradigm of inattentional blindness research (Rensink et al., 1997). This paradigm challenges participants to detect a change between an original and manipulated scene when quickly flickered back and forth between the two and separated by a brief flash (as long as a slow blink). Under these conditions, participants struggle to detect that anything has changed at all. Instead, participants are strongly predisposed to assume constancy and report that nothing has changed between the two scenes. These paradigms have been used to promote a model of memory wherein significantly less information about our environment is represented in the brain than was believed prior to the discovery of change blindness, and rather that we depend on the environment itself to serve as an external memory (O’Regan et al., 1999; O’Regan, 1992). As a result of this interpretation, these experiments have tended to be characterized as purely attentional and not as working-memory experiments at all (Rensink et al., 1997; Simons & Levin, 1997). There is an inherent working memory component of the flicker paradigm, however, as participants are tasked with comparing the current iteration of a scene with their memories of the scene only a moment before. Furthermore, participants are usually able to detect the object of change eventually, suggesting that something is being compared to the immediately perceived scene, and thus some representation of the scene must exist in memory for comparison. The discovery of distinct item capacity limits to working memory (Luck & Vogel, 1997) usefully informs how change may be detected in flicker paradigms, as participants may be holding only a small subset of items in their working memory between fixations for comparison. Given that this paradigm uses complex, naturalistic scenes with many objects that could have potentially undergone a change, this capacity limit accurately predicts that participants would take many comparisons before they eventually got lucky and select the right objects for comparison.

Although the flicker paradigm offers a striking demonstration of change blindness, the paradigm’s lack of flexibility makes it less than ideal for further explorations of the phenomenon of change blindness. For one, because the flicker paradigm relies on ambiguous targets of change in a scene, complex naturalistic scenes with many potential targets of change are regularly used, thus presenting a set of possible change targets well above the capacity of working memory. Furthermore, because only one item (the target) can change in a flicker task, it is not possible to compare trials in which the majority of the scene remains constant with trials in which some part of the scene underwent some irrelevant changes. While it is conceivable to design a version of the flicker paradigm that addressed these issues, there already exist other tasks that could easily be adapted to answer these questions. One appealing alternative is the change-detection working-memory test designed by Luck and Vogel (1997), which is a simple and flexible paradigm designed explicitly to test capacity limits in working memory. In this task, participants are instructed to remember a small set of memory stimuli presented on a screen and to report whether one of them has changed after a brief retention interval. This task was further adapted by Wheeler and Treisman in 2002 to observe change detection for a variety of object features as well as for feature combinations. This paradigm offers several advantages over the flicker paradigm while still concerning itself with the basic task of change detection: Firstly, it offers the opportunity to observe whether change blindness occurs when only a small subset of items are marked as memory targets.

If constancy is strongly influenced by environmental factors, by embedding a simple change-detection task in complex visual environments that do not change, participants should become worse at detecting when a change has occurred to one of the memory items (i.e., what is observed in change-blindness experiments). Furthermore, by instructing participants explicitly that only the memory targets are relevant to the change-detection task, the backgrounds become available for manipulation. This opens the door to compare change-detection accuracy between trials in which the environment remained entirely stable to trials in which significant visual changes have occurred, a comparison that the flicker paradigm is incapable of making due to the ambiguous nature of the change target. It may be the case that constancy bias persists despite this added environmental activity, indicating that regardless of global events we default to assuming that objects outside our attention have remained constant. Alternatively, this salient environmental activity may represent compelling evidence that unattended items have undergone a change, resulting in the disappearance of constancy bias or even a reversal of the bias effect as background changes may be misattributed as stimuli changes, resulting in false memories and illusory changes to the memory stimuli.

To test these conditions, we chose to model our task on that used by Wheeler and Treisman in their 2002 paper. In their experiments, Wheeler and Treisman tested a variety of stimuli and change types to investigate working-memory capacity for feature conjunctions. Wheeler and Treisman’s paradigm closely copied that of Luck and Vogel except that in some experiments, the stimuli were a selection of shapes instead of colored boxes, and the types of changes that occurred in a trial were more numerous than in Luck and Vogel’s paradigm. This allowed them to observe differences in change detection for features versus binding changes. We hoped that by adopting their paradigm we would get a similarly rich view on the influences of environment of various types of change detection. Furthermore, by using shapes instead of color stimuli as memory targets, we were free to use colored backgrounds as our environmental manipulation. For our task participants needed to remember the identies and locations of four shape stimuli, which were presented on complex, multicolored backgrounds that could change at some point between learning and recall. By using four memory items, we presented participants with a challenging memory task, but one that was significantly easier than change detection in a complex, natural scene. In essence, our paradigm allows us to observe whether working memory is influenced by environmental factors, or whether working memory was entirely insensitive to irrelevant, incidental environmental activity.

## Experiment 1

### Methods

#### Participants

We recruited 16 participants^1^ (11 females, age 18–29 years) through advertisements around the Uithof campus of Utrecht University as well as online through various paid participant recruitment websites. These participants were compensated €10 for an hour and a half of participation. All participants had normal or corrected-to-normal vision and all participants gave informed consent before beginning the experiment.

#### Stimuli

Our experiment took place in a room with black walls and with the lights turned off. Visual stimuli were presented on a 54.61 cm, 1,920 × 1,080 resolution, 60 Hz LG Flatron W2261 monitor connected to a Macintosh MacPro version 10.10.5 running the Python library program PsychoPy (v1.85.6) (Peirce, 2009). Participants were provided with a chin rest situated 60 cm from the test monitor.

Our stimuli were based on those used by Wheeler and Treisman in their 2002 paper (Experiment 4). Memory targets were a set of nine possible shape stimuli. The shapes were chosen due to their familiarity and were: a circle, triangle, diamond, trapezoid, pentagon, arrow, hexagon, star, and cross. All shape stimuli were approximately 2 visual degrees in size and dark gray in color. The locations that the shapes could appear were pseudo random in that the test screen was divided into an invisible 3 × 4 grid of evenly spaced locations approximately 5 cm apart from one another and the screen edge. To make the shapes seem like they were not being placed on a grid, a random (x, y) value was added to each position when it was selected to hold a shape, making it seem that shapes appeared at random locations in space.

**Fig. 1:**
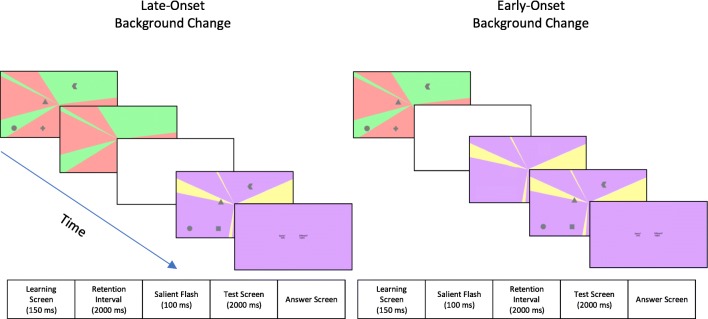
The experimental flow for the two onset conditions

While many change-detection working-memory tasks use color stimuli, by using shape stimuli we were able to vary background colors without overlapping with the relevant memory features. The background colors used were all light in shade and highly luminous to ensure that the dark shapes were suitably visible regardless of what color was used. Colors were selected for their discriminability and familiarity. The colors used were red (10 candelas), blue (11.4 cd), green (16.1 cd), yellow (18.5 cd), pink (10.8 cd), purple (10.1 cd), orange (12.4 cd), and gray (16 cd). The background design we used was a two-colored vanishing point design where the screen was divided into four colored triangles converging at the centre of the screen (see Fig. 1 for an example).

#### Design and procedure

The experiment lasted approximately one and a half hours including five breaks, with a break occurring approximately every 10 min. The length of the break was left up to the participant, though they were made aware that longer breaks would lead to later finish times and as a result many participants took short breaks or even chose to periodically skip breaks.

The experiment began with both a verbal description of the experiment as well as a comprehensive written description on the computer that included visual demonstrations of the types of stimuli and changes to expect. The experiment consisted of 576 trials broken up into six blocks of 96 trials each, with a break between each block. (A breakdown of the number of trials for each condition is included in Table 1.)

**Table 1:** Accuracy (% correct) and trial count (per-participant) across no-change trials and the three change-trial types separated between the two onset conditions

	Late-Onset Background Change		Early-Onset Background Change		Trials (Per-Participant)
	Same Background	Different Background	Same Background	Different Background	
No-Change	78.47	71.81	87.15	86.28	288
Position	92.71	93.75	90.63	89.06	96
Binding	58.85	64.84	52.86	53.65	96
Feature	50.26	56.24	40.36	45.83	96
Trials (Per-Participant)	144	144	144	144	576 (Total)

Notice that position changes were consistently easy to detect and were uninfluenced by the various experimental conditions

The progression of a standard trial is given in Fig. 1 and was based on the experimental design of Wheeler and Treisman (2002), which in itself was based on the paradigm used by Luck and Vogel (1997). Each trial began with a learning screen in which a four-shape array of memory targets were presented on a multi-colored background. The learning screen was presented briefly for 150 ms, after which the memory items would disappear, and a retention interval of 2 s would follow. We chose to use a relatively long retention interval so that we could be absolutely confident that image after-effects were not useful in our task.^2^ During the retention interval, a black fixation cross would be centrally presented. Participants were never explicitly told to fixate on the cross. Following the retention interval, the test array would be displayed, consisting of four shapes that could either exactly match the learning screen or differ in one of three ways. The three types of change possible were taken from Experiment 3 of Wheeler and Treisman’s (2002) paper and were: position, feature, or binding changes. In position-change conditions, one of the shapes would move to a new coordinate on the screen. In feature-change conditions, one of the shapes would change to a new shape not previously included in the test set. In binding-change conditions, two of the shapes would swap positions, thus the shape and position sets were the same, but participants had to identify that the binding of these characteristics had changed. Half of all trials featured one of the three types of changes, and through the experiment a participant would encounter each type of change 96 times. For the other half of the trials, the test shape array would exactly match the feature and position combination as in the learning screen, and were thus no-change trials.

The test screen would be presented for 2 s, after which the test stimuli would disappear, and the subsequent answer screen would prompt participants to indicate whether they believed it was a change or no-change trial. Participants indicated their answers using the left and right arrow keys corresponding to “no-change” and “change,” respectively. As is normal in change-detection working-memory tasks (e.g., Luck & Vogel, 1997; Wheeler & Treisman, 2002), our task was a two-alternatives forced-choice task, meaning participants were forced to provide their best guess in cases when they were unsure of the correct answer. This experimental design is visualized in Fig. 1.

Additionally, on half of the trials, the background would remain constant throughout the trial, and in the other half it would change sometime between learning and test screens. Introducing background changes presented us with the interesting question of when in a trial should the background change. We treated this question as non-trivial as the onset timing of the background change can have a positive or negative effect on the change-detection tasks (Baker & Levin, 2015).

We therefore chose to use two onset timings for the background changes: either the background could change early in the trial (early-onset) or late in the trial (late-onset). In early-onset trials, the change would occur immediately after the learning stimuli disappeared, and thus participants would see the new background during the 2-s retention interval. In late-onset trials the change would occur immediately before the test stimuli were presented, so participants would be confronted with the test stimuli and a new background in the same moment. If participants were only sensitive to the identity of visual environments as the same or different, we did not expect the different onsets to exhibit different results. However, if participants were sensitive to when a change occurred in temporal proximity to stimuli learning or recall, then we expected this manipulation to illicit different patterns of results.

A last element of our experimental design was the inclusion of a salient white flash on all trials. We chose to include a white flash so that there would be some level of environmental activity in background constant trials. If we had not included this activity, there would have been an additional dimension of difference between our background conditions as in background-change trials there is necessarily always background activity in the form of the background change, but on background-same trials there need not be any environmental activity at all, creating a potential confound for any observed results. In order to ensure that in all trials there would be some level of background activity, we chose to add salient visual activity to all trials in the form of a 100-ms white flash. This flash would occur immediately before the background change, corresponding to the trial’s onset-timing condition. On trials in which the background remained the same, the only difference between early- and late-onset trials was when this salient flash occurred.

### Results

**Fig. 2:**
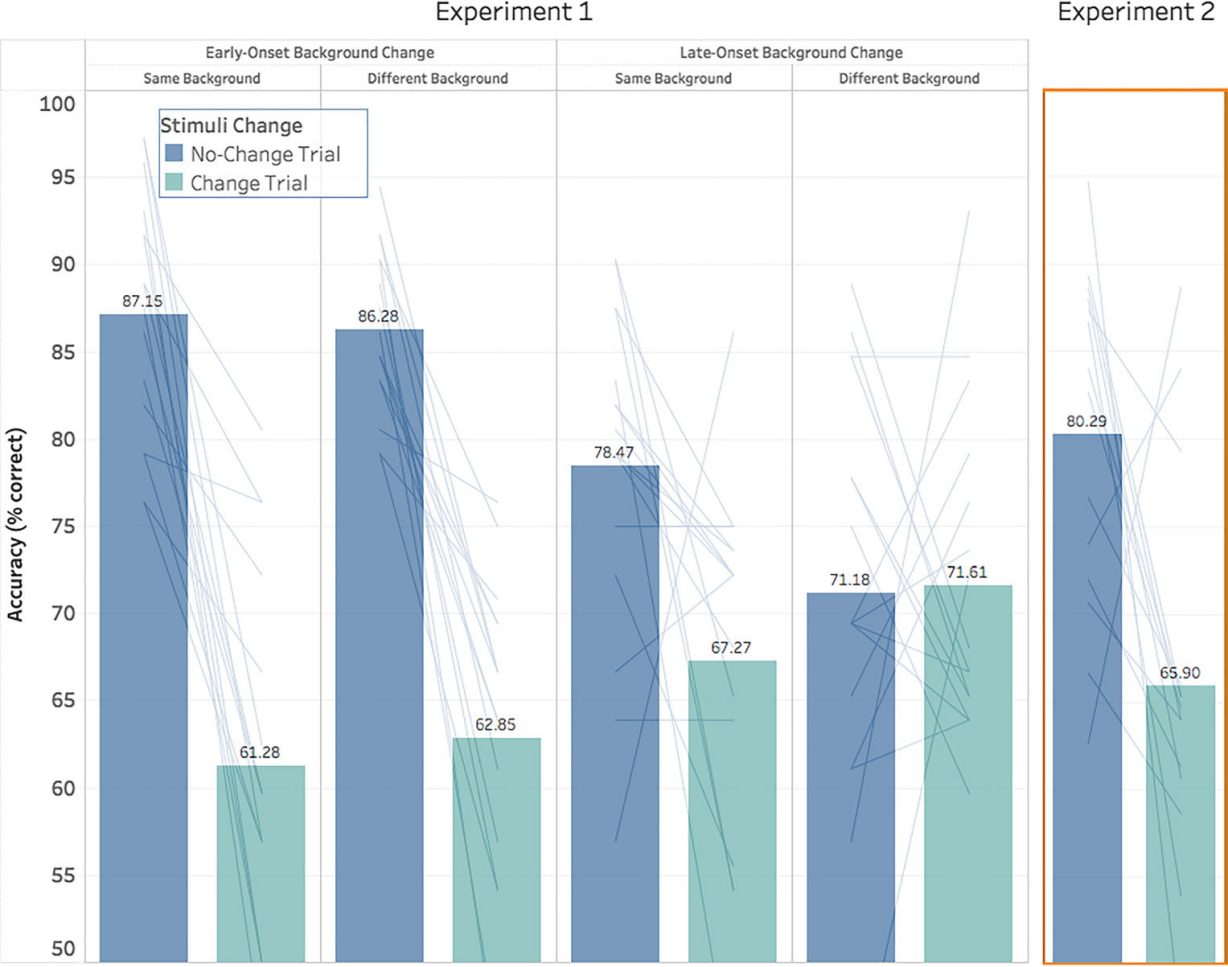
Experiment 1 (**Left**): Shown is the change detection accuracy between change and no-change trials across the two onset conditions and two background conditions. In early-onset trials, there was no difference between background conditions, as participants were consistently biased towards change rejection. In late-onset trials, participants were sensitive to background condition as they showed a bias towards change rejection when the background remained constant but showed no bias towards change rejection nor detection when backgrounds changed immediately before stimuli presentation. Experiment 2 (**Right**): Shown here are the results of our second experiment where backgrounds remained a constant neutral gray across all trials. These results closely replicate the pattern of bias observed in Experiment 1 caused by constant backgrounds. It may be noted that the results do not perfectly match the early-onset background-same trials as would be expected, but rather seem to fall between the early- and late-onset background same results. This difference may suggest that the increased attentional recruitment cause*d* by salient, multicolored backgrounds of Experiment 1 had an effect on the consistency of the bias effect [*g*r*a*y lines in these figures indicate changes in individual participant scores between conditions]

All results were analyzed using JASP, an open-source data analysis software similar to SPSS (JASP team, 2018). A 2 × 2 × 2 repeated-measures analysis of variance (ANOVA) was conducted taking onset timing (early/late), background (same/different), and stimuli (change/no-change) as factors and accuracy as the dependent variable 3 These factors are visualized in Fig. 2.

Main effects were found for onset timing (*F*(1,15) = 4.824, *p* = 0.044, *η*^*2*^ = 0.243) and stimuli conditions (*F*(1,15) = 61.42, *p* < 0.001, *η*^*2*^ = 0.804), but not for background (*F*(1,15) = 1.174, *p* = 0.296). Participants were more accurate in early-onset trails than late-onset trials (74.39% and 72.14% accurate, respectively). The main effect of onset timing can be characterized as a preference by participants for test stimuli to be presented without additional visual activity, though this preference only amounted to a 2% increase in accuracy. Participants were much more likely to correctly mark a no-change trial than a change trial (80.77% and 65.76% accuracy, respectively). This 15% difference in accuracy can be characterized as a general bias to perceive stimuli as unchanging.

Several interactions were observed between onset and stimuli conditions (*F*(1,15) = 16.74, *p* < 0.001, *η*^*2*^ = 0.527), as well as background and stimuli conditions (*F*(1,15) = 19.68, *p* < 0.001, *η*^*2*^ = 0.568). Furthermore, an interaction between all three factors was found (*F*(1,15) = 5.628, *p* = 0.031, *η*^*2*^ = 0.273). No interaction was found for onset and background conditions (*F* < 1).

To simplify our illustration of these interactions, we have broken down our 2 × 2 × 2 repeated-measures ANOVA into two separate 2 × 2 repeated-measures ANOVAs by separating the onset-timings conditions while keeping background and stimuli change conditions as factors. We chose to separate our analysis based on onset timing as they nicely illustrate very different participant behaviors when stimuli were or were not presented with incidental visual activity. These differences are shown in Fig. 2.

#### Early-onset trials

For trials in which the background change and salient flash occurred early in the trial and were separated from the test stimuli by several seconds, a main effect of stimuli condition was observed (*F*(1,15) = 67.12, *p* < 0.001, *η*^*2*^ = 0.817), but not for background condition (*F* < 1). Furthermore, no interaction between the factors was found (*F*(1,15) = 1.154, *p* = 0.30). When the background change onset was early in the trial, participants were insensitive to whether the test stimuli were presented on the same or a different background to the one they learned on. Participants were also consistently and strongly biased towards reporting that no change had occurred, where no-change trials were correctly identified 86.72% of the time while only 62.07% of stimuli change trials were correctly identified. This effect was also very consistent, as all participants showed this bias towards change rejection.

#### Late-onset trials

A very different pattern of results was observed when either backgrounds changed or the salient white flash occurred immediately before the test stimuli were presented. While no main effects were found for stimuli or background conditions (*F*(1,15) = 1.715, *p* = 0.210 and *F*(1,15) = 1.329, *p* = 0.267 respectively), an interaction between these factors was observed (*F*(1,15) = 16.28, *p* = 0.001, *η*^*2*^ = 0.521). When backgrounds changed immediately before stimuli presentation, participants were more likely to correctly identify a change trial (71.61% accurate for background change trials and 67.27% accurate for background same trials); when backgrounds remained the same, participants were much more likely to perceive a trial as a no-change trial (78.47% accurate for background same trials and 71.61% accurate for background change trials). Because overall accuracy remained the same between these trial types (68.91% and 66.93% accurate for background same and background different trials, respectively), this difference can be attributed to changes in guess behavior, where when participants were unsure about whether a change had occurred, their decisions were strongly influenced by background behavior.

Additionally, participants were consistently less biased towards change rejection in late-onset trials than early-onset trials, even when the backgrounds remained constant. This can be observed by comparing accuracy on the two stimuli conditions between onset conditions on trials in which the backgrounds remained the same. Late-onset background same trials were significantly less accurate on no-change trials (*t*(15) = -4.600, *p* < 0.001), and significantly more accurate on change trials (*t*(15) = 2.805, *p* = 0.013). Because the only difference between early- and late-onset trials in which the background remained constant was the timing of the salient flash, the fact that participants were less biased towards change rejection in late-onset trials can be taken to show that the mere presence of some environmental visual activity (the flash) immediately before stimuli presentation affects whether the stimuli will be perceived as the same or different.

Additionally, the three different change types were compared to see how change detection accuracy varied depending on what type of change occurred in a trial. A repeat-measures ANOVA taking the three types of changes as factors showed a significant difference in accuracy depending on which type of change had occurred in a trial (*F*(2,15) = 180.46, *p* < 0.001, *η*^*2*^ = 0.923). Feature-change trials were consistently the hardest to detect and were only above 50% accuracy in one test condition (late-onset trials in which the background did change). Binding-change trials were consistently easier to detect and were on average 9% more accurate across all experimental conditions. Position-change trials, however, were consistently easy to detect, with participants correctly identifying position changes an average of 91.67% of the time they occurred, and did not vary significantly between background or onset conditions (*F*(1,15) = 2.411, *p* = 0.079). The behavior of specific stimuli-change conditions across background and onset conditions can be seen in Table 1.

It is worth observing that 50% accuracy on a four-shape-change detection task suggests that participants were consistently able to remember about two of the four items. One useful measurement for working memory capacity is Pashler’s K measurement (Pashler, 1988), which rates working memory capacity based on change-detection accuracy in small memory arrays to produce a rough estimate of memory capacity. The average K score in our experiment was 2.30 items.^4^

## Experiment 2

Change-detection tasks have been a standard in the measurement of working memory, both with colored stimuli (Luck & Vogel) and with shape stimuli (Allen et al. 2012; Awh et al., 2007; Wheeler & Treisman, 2002). Despite this, rarely have participant-response biases been reported, nor has the effect of presenting stimuli on neutral backgrounds been investigated. The findings of Experiment 1 suggest that a change detection paradigm using stable backgrounds should result in a bias towards change rejection as participants tend to perceive these objects as constant (as is the case in change-blindness tasks). To test this prediction, a second experiment was conducted mirroring exactly the stimuli, apparatus, and conditions of the first except that backgrounds never changed and remained a constant gray color throughout all trials.

### Methods

#### Participants

Fifteen new participants (eight females, age 19–28 years) were recruited in the exact same way as in Experiment 1. One participant was excluded after it was revealed during debriefing that they believed the experiment had involved deception and that every trial had been a change trial.

#### Stimuli, design, and procedure

The stimuli and experimental design were exactly the same as in Experiment 1 with the exception that the backgrounds never changed in this new experiment. Because we did not test background-change trials, and because we matched the number of no-change trials between this experiment and Experiment 1 (288 trials, this version of the experiment was significantly shorter than Experiment 1, 45 min rather than 1 h 30 min).

### Results

Our observed results closely matched those observed in the background same condition of Experiment 1. Participants were significantly more likely to report that the shapes had not changed on all trials, and were much less likely to successfully detect a change in stimuli (80.29% and 65.90% accurate on no-change and change trials, respectively; *t*(13) = 2.974, *p* = 0.011). The average K score was 2.30, exactly the same as that observed in Experiment 1. These results are shown in Fig. 2.

### Discussion

The aim of this study was to test whether constancy bias could be observed and measured in an adaptation of a simple working-memory change-detection task (Luck & Vogel, 1997; Wheeler & Treisman, 2002), thereby offering a means for studying change blindness using a more flexible task than the commonly used flicker paradigm (Rensink et al., 1997). We observed that participants were strongly biased to perceive stimuli as constant and unchanging when the test stimuli were presented on the same backgrounds as they were learned on. Furthermore, participants were also biased towards perceiving constancy when they had the opportunity to become familiar with a new background before test stimuli were presented, suggesting that the effect of bias was not a top-down matching of stimuli and context, but rather was sensitive only to the amount of visual activity that occurred immediately before stimuli presentation. This activity was factored into the estimate as to whether unattended objects were likely to have undergone a change, and this change in bias was reflected in the changing accuracy on stimuli change and no-change trials.

The assertion that participants were only sensitive to the amount of visual activity immediately before stimuli presentation is further supported by the observation that the constancy bias was reduced on background-same trials when the salient flash had a late onset. For trials that did not feature a background change, the only difference between early- and late-onset conditions was when during the trial the salient flash occurred. The fact that the presence of a flash immediately before stimuli presentation led to a weaker constancy bias indicates that participants were in tune to the amount of visual activity at stimuli presentation, even when it was only a short flash, and adjusted their bias to account for this activity. Furthermore, the only conditions that did not show any constancy bias were late-onset background-change trials, where the most visual activity accompanied the presentation of the test stimuli.

Clear differences in behavior were also observed between the three change types, where position changes were consistently very easy to detect and entirely insensitive to experimental conditions. This was in contrast to feature and binding change trials, which were both much harder to detect and varied in accuracy along the different experimental conditions. While feature-location binding was used to test binding behavior in Wheeler and Treisman’s 2002 paper (Experiment 3), numerous theories of object perception suggest a separation between the mechanisms responsible for encoding spatial and feature information, including Treisman’s own Feature Integration Theory (Treisman & Zhang, 2006; Treisman 1998). The ease with which spatial changes were detected and the insensitivity of these trials to the various experimental conditions we utilized further supports a version of feature integration theory that treats spatial-feature and feature-feature binding as separate processes. This pattern of results therefore contributes to a robust body of evidence for a dissociation between feature and spatial working memory (Awh & Jonides, 2001; Baddeley, 1974; D’Esposito et al., 1998).

Interestingly, binding changes were consistently easier to detect than feature changes, a pattern of results that was not predicted based on Wheeler and Treisman’s findings in their 2002 paper from which our experimental paradigm borrowed heavily. Wheeler and Treisman suggested that object binding should be the most difficult type of change to detect (Wheeler & Treisman, 2002). Rather, we found that feature changes were the most difficult. The reason for this pattern of results may have to do with the fact that participants were not regularly able to remember all four items in our stimuli set, as evidenced by their low feature change detection scores. Testing binding changes implicitly assumes that participants have properly encoded all memory items. Under these conditions, binding changes are said to be more difficult to detect because it is not just the features that need to be remembered but also their specific combination. In conditions in which one or more of the items are not remembered, behavior on binding change trials is very different. If only a subset of items are properly remembered, and features are switched between one remembered and one unremembered stimulus, then perceptually these trials will be indistinguishable from trials in which an item simply changes to a new feature not previously present in the item set at all. Essentially, binding changes are only difficult if all items are properly encoded but are easier than feature changes if only a part of the memory set is encoded, as in feature-change trials only one item undergoes a change, whereas in binding-change trials, two items undergo a change.

Following the completion of Experiment 1, we wanted to know how much of the observed behavior was due to the different background conditions and how much was inherent in our experimental design. Answering this question would allow us to hypothesize how generalizable the current results are to other change-detection paradigms used to measure working memory (e.g., Luck & Vogel 1997; Wheeler & Treisman, 2002). To this end, we ran a second experiment where we exactly replicated our first experiment except that the backgrounds never changed, but remained the same, neutral shade of gray across all trials. Under these conditions, we saw a pattern of results very similar to those observed in Experiment 1 when the backgrounds remained constant throughout a trial; participants were again significantly biased to perceive the shapes as unchanged when presented on stable, unchanging backgrounds. These results suggest that bias to perceive objects as constant when presented on unchanging backgrounds may be generalized beyond our multicolored background conditions and is thus a general inattentional principle.

Because our task was a two-alternatives forced-choice task, it is worth considering how much of our observed effect was due to guess-work caused by the stimulus set being larger than the natural working-memory capacity of our participants. With an average Pashler’s K score of 2.3 items from our experiments, participants consistently would have had to guess whether one or two of the items had changed on a trial. The resulting bias can thus be accounted for as differences in guess behavior, where environments influence perception in situations of uncertainty. We take this to be a plausible explanation of our results, though the answers provided still represent the perceptual beliefs of participants, and the biasing effect of background was invisible to the participants.

Working memory is a critical tool in change detection; if we have a clear memory of an item then we will not struggle to detect a change to that item (Luck & Vogel, 1997). However, given the severely limited nature of working memory, it is rarely if ever the case that we have properly remembered all the items in our environment, a state of affairs necessitating heuristics, such as constancy, to account for the likely behavior of these unmemorized items. Predicting when an item outside our attention has likely undergone a change is one basic function of inattentional vision, and these predictions are aided by the observation that changes tend to be accompanied by the occurrence of significant visual activity. It is thus the case that when significant visual activity has occurred, we recognize that constancy is not an apt assumption, and instead unattended or unmemorized stimuli are treated as if they were equally likely to have changed as they are to have remained the same. The inattentional principle of constancy can therefore be summarized as such: ‘If visual activity is low, assume all items outside of our attention are remaining constant.”

Despite the fact that inattentional visual processes govern the majority of the visual information that enters our eyes, researchers have tended to ignore inattentional visual processes, instead preferring to study the behavior of objects in our immediate attention. Unattended visual information is not presented in a raw, unstructured way, but instead is represented as coherent objects obeying rules and principles in the same way that attended visual objects do. We are able to comfortably ignore such a large part of our visual environment exactly because inattentional principles are so efficient at governing this ignored information, forming predictions about their behavior while outside of our focus of attention and informing us when this information may have become relevant. A critical principle of inattentional vision is that of constancy, or the tendency of objects to remain constant without accompanying visual stimulus. This principle can be easily flipped to say that when something happens to an object in space, it is often accompanied with salient visual activity, an easy flag for attentional relevance. Without this flag, though, the simple principle of constancy is the guiding heuristic, and we will remember and react to these unattended items as if they were constant and unchanging.

#### Open Practice Statement

The data and experimental code are available upon request. None of the experiments were preregistered.

## Footnotes

1 We chose to use a relatively small sample size as this experiment was heavily piloted as part of the first author’s Master’s Thesis. The effect size was thus approximately known, and the sample size of 16 was deemed appropriate. This study was not pre-registered.

2 During piloting, some participants reported lingering afterimages caused by viewing a strong computer monitor in a dark room. We wanted to be sure these afterimages were no longer present as they could be used to verify whether shapes had changed positions.

3 We chose not to analyze scores using d` or A` detection theory measures as bias was shown to vary greatly between conditions, a variation that sensitivity scores fails to capture (Allen et al., 2006; Pastore et al., 2003). A separate analysis of bias scores using Grier’s nonparametric B`` score (Grier, 1971) was conducted and replicated the pattern of results exactly. For clarity and to avoid redundancy, this analysis has not been included.

4 It may be observed that a K-score of 2.3 is quite low; normally K-scores on a four-item task should be in the neighborhood of 3–3.5. The lower sores observed in our experiment may have been due to the fact that we used shape stimuli rather than the more commonly used color stimuli (the reasons for this choice are given in the methods section) and shape stimuli have regularly been shown to be harder to remember than color stimuli (Allen et al., 2012; Wheeler & Treisman, 2002).
